# Efficacy and safety of CD22-specific and CD19/CD22-bispecific CAR-T cell therapy in patients with hematologic malignancies: A systematic review and meta-analysis

**DOI:** 10.3389/fonc.2022.954345

**Published:** 2022-12-29

**Authors:** Lili Li, Luqin Wang, Qinhua Liu, Zhonghui Wu, Yulong Zhang, Ruixiang Xia

**Affiliations:** ^1^ Department of Hematopathology, The First Affiliated Hospital of Anhui Medical University, Hefei, China; ^2^ Department of Bioinformatics, Precedo Pharmaceuticals Co. Ltd., Hefei, China

**Keywords:** immunotherapy, chimeric antigen receptor t-cell therapy, hematologic malignancy, CD22, CD19/CD22-bispecific, meta-analysis

## Abstract

**Background:**

CD22 single and CD19/CD22 bispecific targeted chimeric antigen receptor T (CAR-T) cell therapy are promising immunotherapy modalities for the treatment of hematologic malignancies. The aim of this study was to assess the efficacy and safety of CD22 and CD19/CD22 targeted CAR-T cell therapy by summarizing the existing evidence.

**Methods:**

Electronic databases including PubMed, Embase, and Scopus were comprehensively searched from inception up to November 30, 2022. Pooled response rates and minimal residual disease (MRD) negative response rates, cytokine release syndrome (CRS) rates and neurotoxicity rates were calculated. Subgroup analysis was performed based on the type of immunotherapy.

**Results:**

Ten clinical studies including 194 patients with hematologic malignancies were included after a systematical screening of literature. The pooled complete response (CR) rates of CD22 and CD19/CD22 CAR-T cell therapy for relapsed or refractory B-cell lymphoblastic leukemia (B-ALL) were 0.75 (95% CI: 0.60 - 0.88) and 0.87 (95% CI: 0.76 - 0.96). The overall MRD negative response rates of CD22 and CD19/CD22 CAR-T were 0.54 (95% CI: 0.42 - 0.66) and 0.91 (95% CI: 0.47 - 0.88). Pooled CRS rates of CD22 targeted and CD19/CD22 targeted immunotherapy were 0.92 (95% CI: 0.82 - 0.98) and 0.94 (95% CI: 0.82 - 1.00), respectively.

**Conclusion:**

Both CD22 and CD19/CD22 CAR-T immunotherapy demonstrated favorable efficacy and acceptable adverse events in the treatment of hematologic malignancies. Well-designed and large sample-sized clinical trials are warranted.

## Introduction

High dose chemotherapy and hematopoietic stem cell transplantation are standard curative strategies for patients with hematological malignancies including myeloid leukemia (AML), acute lymphoblastic leukemia (ALL), Hodgkin’s lymphoma and diffuse large B cell lymphoma (DLBCL) (1). For example, treatment options for AML included either intensive chemotherapy with anthracycline and cytarabine-based regimens (7 + 3) or lower intensity regimens including hypomethylating agents or low dose cytarabine, followed by either allogeneic stem cell transplant or consolidation chemotherapy (2). Treatment for pediatric ALL typically consists of induction therapy with steroids, vincristine, and asparaginase with or without anthracycline, followed by multi-agent consolidation including high-dose methotrexate and re-induction therapy (3). Nevertheless, sufficient efficacy occurred temporarily due to disease relapse following treatment (4).

Interestingly, immunotherapy has been extensively investigated for decades in the treatment of almost all types of hematologic cancers (5). Chimeric antigen receptor (CAR) T cell therapy is one of the promising immunotherapy approaches (6, 7). CARs are genetically engineered proteins that manipulate the antigen-recognition ability of antibodies and the effector functions of T cells (8). A CAR is composed of three pivotal domains including an extracellular antigen recognition domain, a transmembrane domain, and an intracellular T cell activation domain (4, 6). T cells were collected from the patient or the donor, amplified in a bioreactor and modified to express a specific CAR before injection into the patient (9). Early clinical data have generated considerable promise, and it is reasonable to speculate that CAR-T based immunotherapy can fundamentally change the existing treatment paradigms of B-cell malignancies (10–14). Furthermore, CD19 directed CAR-T cell therapies namely tisagenlecleucel, axicabtagene ciloleucel, brexucabtagene autoleucel, and lisocabtagene maraleucel received the FDA approval in recent years (15–17). CAR T-cell therapy, however, is related to notable drawbacks that hinder its development and wide promotion: cytokine release syndrome (CRS), immune effector cell-associated neurotoxicity syndrome (ICANS), B-cell regeneration disorders, and early and late infections (18–21). Of note, signs and symptoms of CRS can vary from mild to fatal (22). Albeit with worldwide exciting clinical data on anti-CD19 CAR-T therapy, relapse after this therapy is associated with poor prognosis and has become an urgent problem to be solved (7, 23). The recurrence of hematologic malignancies partly resulted from the loss of CD19 antigen expression on malignant cells after CD19 CAR-T cell treatment, thus CAR-T with new or dual targets on CD19 and CD22 may address the drug resistance (4).

CD22 CAR-T cell therapy, proposed as an alternative CAR-T method for treating primary or relapsed B-lymphoblastic leukemia (B-ALL), have been demonstrated to be effective in the treatment of patients with B-ALL who are not suitable for CD19 CAR-T cells therapy (24–27). Moreover, CD19 and CD22 bispecific CAR-T therapy *via* transduction of T cells with a bicistronic γ-retroviral vector encoding humanized anti-CD19 and CD22 CARs has also been investigated and revealed elicited clinical outcomes (28). Accumulated number of clinical trials with regard to CD22 single and CD19/CD22 bispecific CAR-T therapy in patients with hematologic malignancies has been performed, nevertheless, the sample sizes and outcomes of these studies are heterogeneous. The aim of this systematic review and meta-analysis was to assess the efficacy and safety of CD22 targeted and CD19/CD22 bispecific CAR-T cell therapies by summarizing the published data and to provide for clinicians with evidence-based references for clinical decision-making and scientific research.

## Methods

The present meta-analysis was performed strictly based on the Preferred Reporting Items for Systematic Reviews and Meta-analyses (PRISMA) statement (29). Informed consent was not acquired because the dataset used in this meta-analysis was derived from published literature.

### Literature search and study selection

Three online databases including PubMed, Embase, and Scopus were systematically searched by two independent authors from inception of the database to November 30, 2022 with articles in English language considered. The references of relevant reviews were manually screened for potentially eligible studies. The following terms and keywords were employed in literature search: CAR-T, chimeric antigen receptor -T cell, CD22, Hematologic Malignancies, Hematologic Neoplasms, Hematologic Neoplasm, Hematologic Neoplasia, Hematologic Neoplasias, Hematologic Tumor, Hematologic Cancer, and Hematologic Malignancy. Two investigators independently performed the study selection, disagreements were addressed through discussion.

Studies fulfilling the following inclusion criteria were enrolled:

1) Clinical studies investigating the efficacy and/or safety of anti-CD22 or anti-CD19/CD22 CAR-T cell therapy in the treatment of hematological malignancies.

2) Outcomes included complete response rate, partial response rate, overall response rate, minimal residual disease (MRD) negative response rate, progression-free survival, overall survival, cytokine release syndrome (CRS) rate and neurotoxicity rate.

3) Literature types included but not limited to research articles in case that complete data could be extracted.

Exclusion criteria were as follows:

1) Patients treated with combinations of CAR-T and other immunotherapies were excluded.

2) Case reports or case series, reviews, meta-analyses, abstracts or correspondence with unavailable data.

3) Citations not in the English language.

### Data extraction and quality assessment

A predesigned table for the variables of data extraction was proposed. Parameters including name of first author, year of publication, country, number of patients, age of patients,percentage of the female, type of malignancies, type of CAR-T and information on outcomes regarding efficacy and safety aforementioned were retrieved by two reviewers, discrepancies were resolved through discussion. If two or more studies covered the same group/subgroup of patients, only the study with the largest sample size or the most complete data was enrolled to avoid duplicates. The Newcastle-Ottawa scale (NOS) was used to assess the quality of the included studies, this scale is a three-domain scale regarding the evaluation of selection, comparability, and outcome (30).

### Statistical analysis

Statistical analyses were performed utilizing the R Foundation for Statistical Computing (Version 4.1.2, Vienna, Austria). Pooled estimates of response rates, adverse effects rates, survival rates with their respective 95% confidence intervals were calculated using the random effects methods considering that most of studies with regard to hematological malignancies were nonrandomized single arm studies. We conducted subgroup analyses to investigate the efficacy and safety according to the type of tumor and type of CAR-T therapies. The I^2^ value was used to assess the magnitude of heterogeneity between included studies. Meta-regression was utilized to evaluate the potential source of heterogeneity. Moreover, the Egger’s test of funnel plot asymmetry were performed to explore the potential publication bias (31). A p value < 0.05 was regarded as statistical significance.

## Results

### Study characteristics

**Figure 1:**
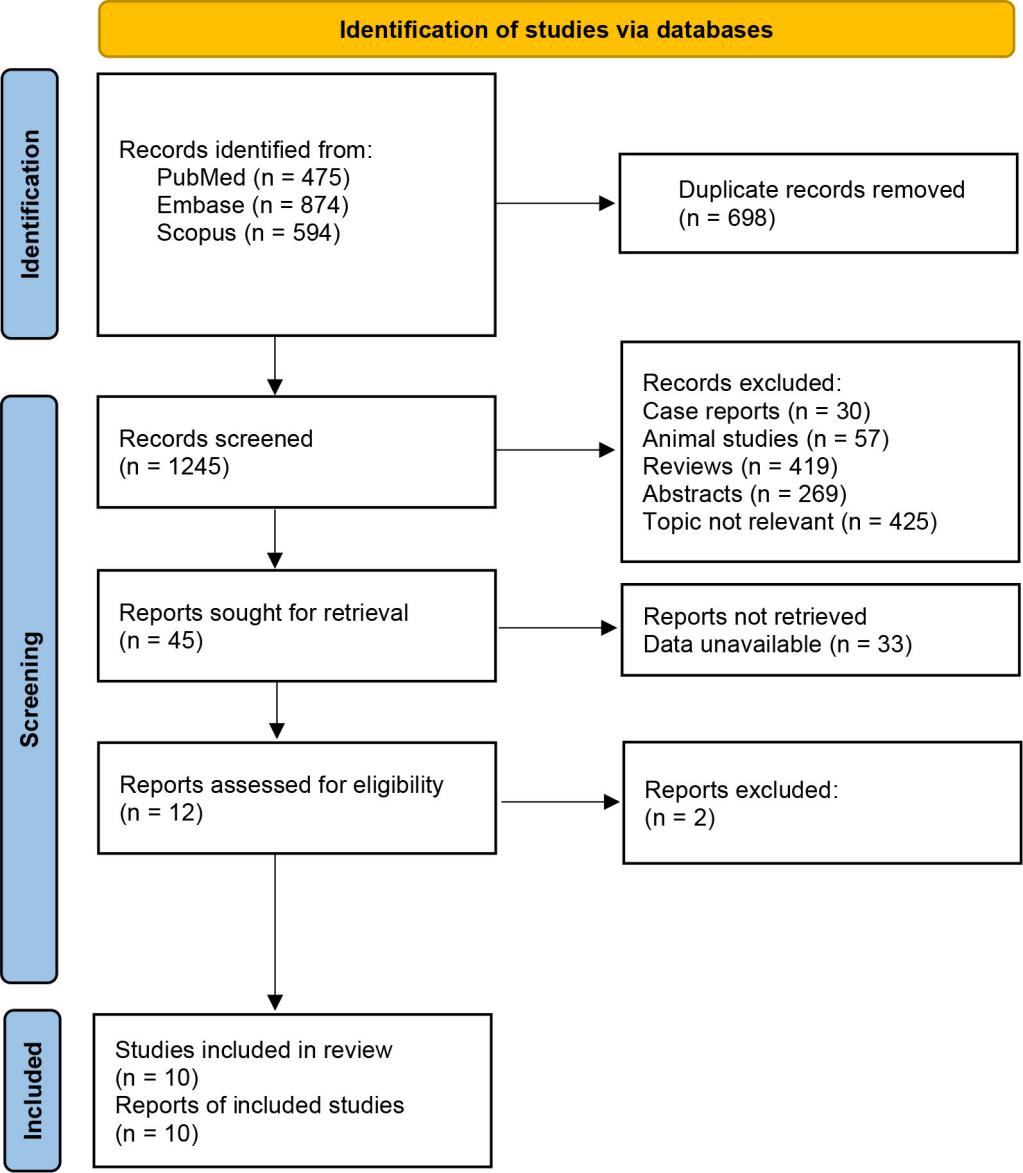
Flowchart of literature search and study selection.

A total of 1943 records were retrieved from the initial database search. After removal of 698 duplicates, 30 case reports, 57 animal studies, 419 reviews, 269 abstracts, 425 topic irrelevant articles, 45 citations were screened in full text reading. Ten clinical studies with 194 patients with hematologic malignancies were eligible for inclusion after full text reading. **Figure 1** shows the detailed flow of literature search. Five included studies were clinical trials investigating anti-CD22 CAR-T cell therapy for relapsed or refractory B-ALL. Five studies assessed the efficacy and safety of CD19/CD22 bispecific CAR-T cell therapy relapsed or refractory B-ALL. **Table 1** reveals the details on characteristics of enrolled studies. The quality of included studies was regarded as moderate to high based on the NOS scale (**Supplementary Table 1**).

**Table 1:** Characteristics of included studies.

First author	Year of publication	Country	Number of patients	Age of patients (yrs)	Female (%)	Type of malignancies	CAR-T type	Reported outcomes
Pan	2019	China	34	10 (1–55)	41	Relapsed or refractory B-ALL	CD22 CAR	CR, MRD negative, CRS, neurotoxicity
Dai	2020	China	6	23 (17–44)	33	Relapsed or refractory B-ALL	Both CD19 and CD22 CAR	MRD negative, CRS, neurotoxicity
Shah	2020	United States	58	18 (4.4-30.6)	NR	Relapsed or refractory B-ALL	CD22 CAR	CR, MRD negative, CRS, neurotoxicity
Wang	2020	China	15	27 (16–65)	53	Relapsed or refractory B-ALL	Both CD19 and CD22 CAR	CR, MRD negative, CRS, neurotoxicity
Cordoba	2021	United Kingdom	15	8 (4–16)	NR	Relapsed or refractory B-ALL	Both CD19 and CD22 CAR	CR, CRS, neurotoxicity
Hu	2021	China	6	49 (26–56)	NR	Relapsed or refractory B-ALL	Both CD19 and CD22 CAR	CR, MRD negative, CRS
Liu	2021	China	27	21 (1.6-55)	NR	Relapsed or refractory B-ALL	CD22 CAR	CR, CRS
Singh	2021	United States	8	NR	NR	Relapsed or refractory B-ALL	CD22 CAR	CR, MRD negative, CRS
Spiegel	2021	United States	17	47 (26–68)	29	Relapsed or refractory B-ALL	Both CD19 and CD22 CAR	OR, CR, PR, CRS, neurotoxicity
Tan	2021	China	8	9 (5–16)	75	Relapsed or refractory B-ALL	CD22 CAR	OR, CR, PR, CRS, neurotoxicity

NR, not reported; OR, overall response; CR, complete response; PR, partial response; MRD, minimal residual disease; CRS, cytokine release syndrome; B-ALL, B-lymphoblastic leukemia.

### Efficacy of CAR-T cell therapy

**Figure 2:**
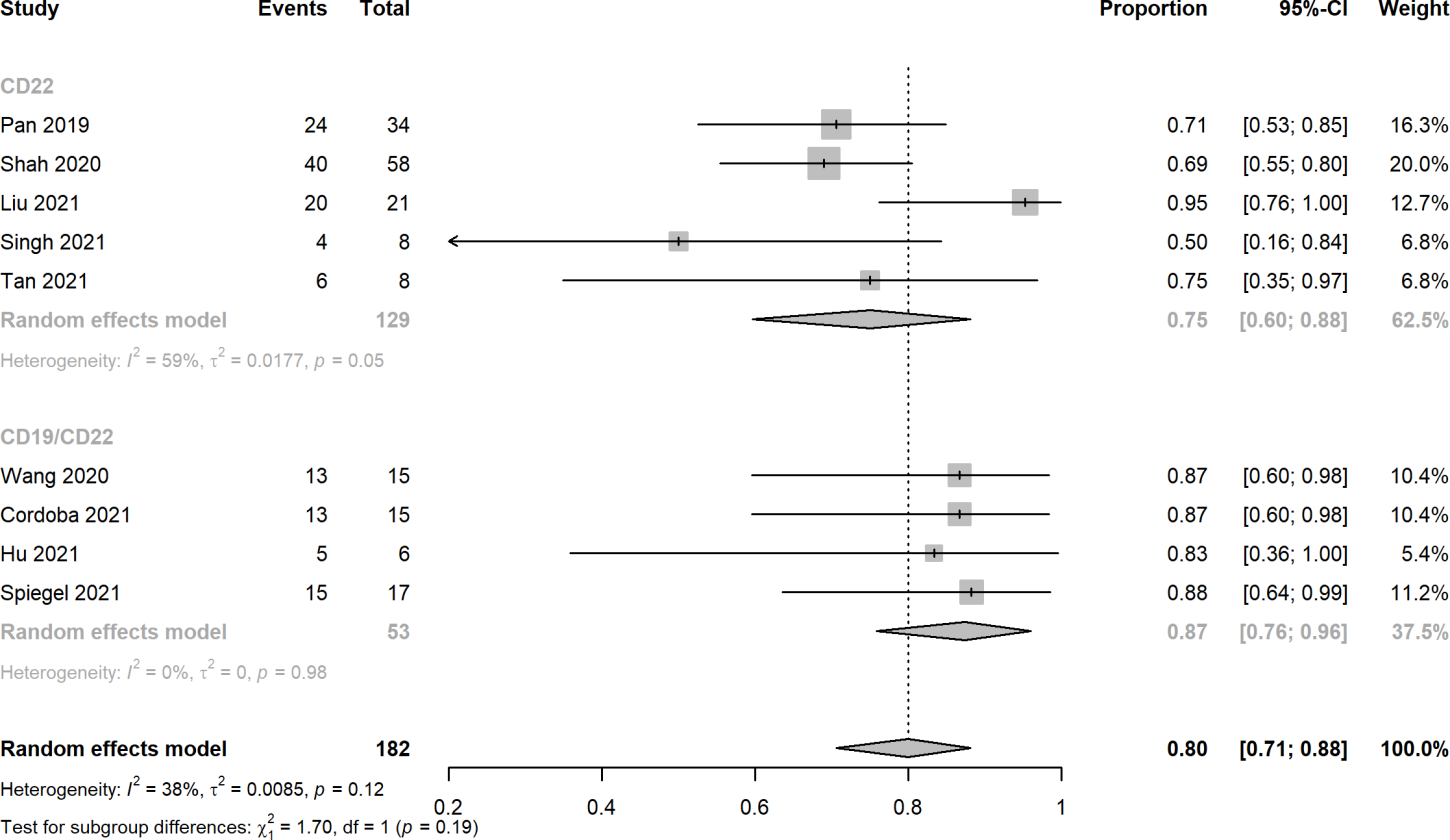
Forest plots of complete response rates of CD22 and CD19/CD22 targeted CAR-T cell therapies for relapsed or refractory B-ALL.

**Figure 3:**
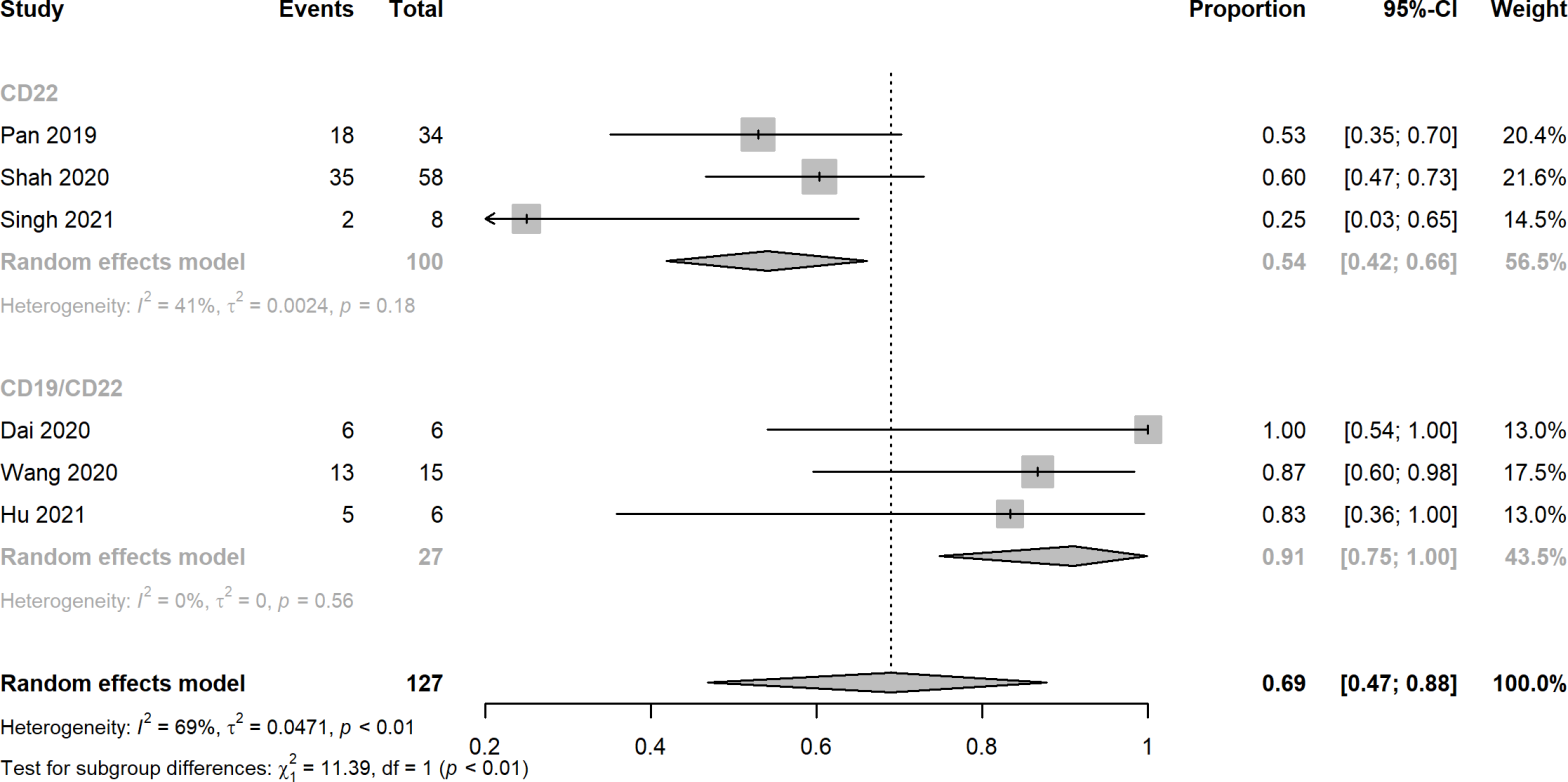
Forest plots of MRD negative rates of CD22 and CD19/CD22 targeted CAR-T cell therapies for relapsed or refractory B-ALL.

Nine studies reported the assessment of treatment complete responses. The overall complete response rates of CD22 and CD19/CD22 CAR-T cell therapies for relapsed or refractory B-ALL were 0.75 (95% CI: 0.60 - 0.88) and 0.87 (95% CI: 0.76 - 0.96), respectively (**Figure 2**). Six studies were evaluated for MRD negative responses, the pooled MRD negative response rates of CD22 and CD19/CD22 CAR-T cell therapies were 0.54 (95% CI: 0.42 - 0.66) and 0.91 (95% CI: 0.47 - 0.88) (**Figure 3**). Two studies reported overall response rates (ORRs), the ORR in the study of Spiegel et al. was 100%, as in the study of Tan et al., the ORR was 87.5%. Besides, results of Shah’s study demonstrated a median overall survival of 13.4 months (95% CI: 7.7 to 20.3 months) and a median relapse-free survival of 6.0 months (95% CI: 4.1 to 6.5 months) for anti-CD22 CAR T cell therapy. Kaplan-Meier survival analysis in Liu’s study manifested overall survival and event-free survival rates of 88.5% and 67.5% at both 12 months and 18 months.

### Safety of CAR-T cell therapy

**Figure 4:**
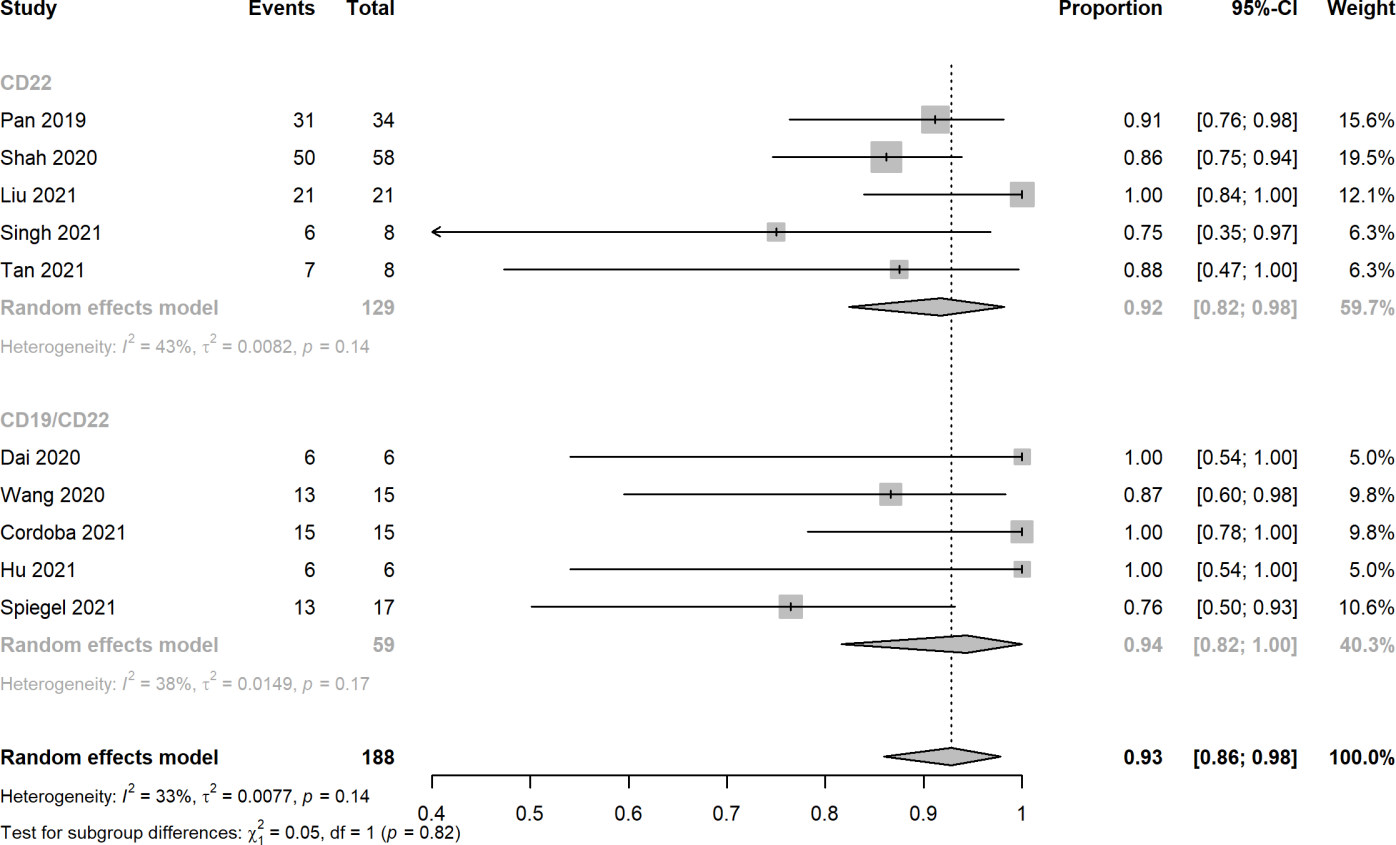
Forest plots of cytokine release syndrome rates of CD22 and CD19/CD22 targeted CAR-T cell therapies for relapsed or refractory B-ALL.

**Figure 5:**
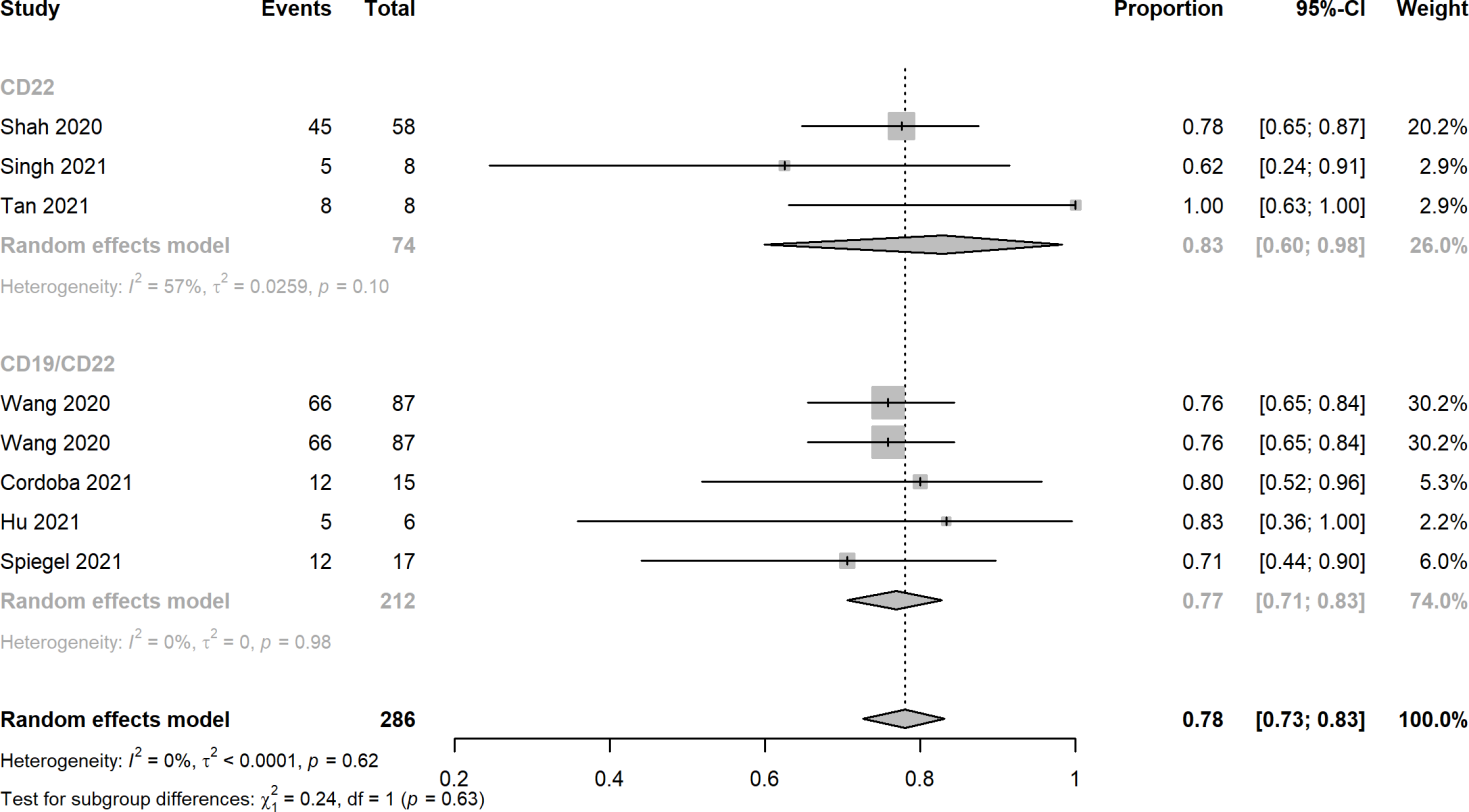
Forest plots of neurotoxicity rates of CD22 and CD19/CD22 targeted CAR-T cell therapies for relapsed or refractory B-ALL.

Ten records reported the rates of CRS, the pooled estimates of CRS rates of CD22 targeted and CD19/CD22 targeted CAR-T immunotherapy were 0.92 (95% CI: 0.82 - 0.98) and 0.94 (95% CI: 0.82 - 1.00), respectively (**Figure 4**). Overall rates of Grade 1 and 2 CRS for CD22 targeted and CD19/CD22 targeted therapies were 0.83 (95% CI: 0.60 - 0.98) and 0.77 (95% CI: 0.61 - 0.90), respectively. In the analysis of neurotoxicity, the pooled rates for anti-CD22 and anti-CD19/CD22 therapies were 0.83 (95% CI: 0.60 - 0.98) and 0.77 (95% CI: 0.71 - 0.83) (**Figure 5**). Low percentage of Grade 3 or above CRS was detected. In the studies of Dai and Cordoba no grade 3 or 4 CRS was observed in any of the treated patients. In Hu’s study, 1 of the 6 patients (16.7%) had grade 3 CRS with hypoxia and required facemask oxygen supplementation (15 L/minute). CRS ≥ Grade3 occurred in 30% (7/23) of patients in Liu’s study. Singh et al. reported one patient had Grade 3 CRS, including significant elevations in serum cytokines compared to the other patients, particularly notable for granulocyte colony-stimulating factor, interleukin-6 (IL-6) and monocyte chemoattractant protein 1. CRS Grade ≥ 3 occurred in 2 patients (5%) in Spiegel’s phase 1 trial. One patient with Grade 3 CRS (12.5%) was reported in Tan’s study. Furthermore, results of Cordoba’s study manifested that one patient 11 relapsed with CD19-negative disease with ongoing CAR T cell persistence > 1,000 copies per μg and B cell aplasia. Hu reported that 3 patients (50%) experienced infections with a severity ≥ grade 3, which included cytomegalovirus reactivation/infection (two cases), bacterial pneumonia (one case), and fungal sepsis (one case), 3 of the 6 patients (50%) experienced cytopenia lasting beyond day 28 after CD19/CD22-targeting CAR-T cells infusion.

### Publication bias

**Figure 6:**
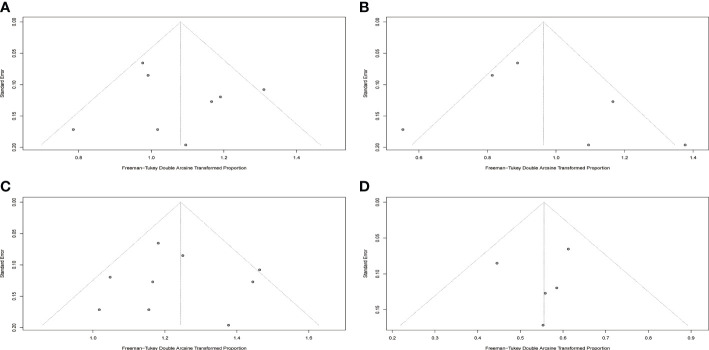
Funnel plots for included studies. **(A)** Funnel plot of complete response rate. **(B)** Funnel plot of MRD negative rate. **(C)** Funnel plot of cytokine release syndrome rate. **(D)** Funnel plot of neurotoxicity rate.

Results of Egger’s tests for publication bias revealed p values of 0.5568, 0.4480, 0.7306, and 0.0595 for CR, MRD, CRS and neurotoxicity which indicated the absence of significant publication bias in included studies. Funnel plots are displayed in **Figure 6**.

## Discussion

Clinical studies with CD19 CAR-T cell therapy have manifested > 70% CR rate in patients with relapsed/refractory B-ALL (14, 32, 33). In addition, a meta-analysis involving 2,172 patients with hematologic malignancies showed an overall response rate of nearly 70% or above. Nevertheless, it is estimated that approximately 50% of CR patients relapsed within 12 months after the primary treatment (7, 23). One of the predominant reasons for the relapse or treatment failure is the mutation or loss of CD19 (26). Another reason may be immune-mediated clearance of murine-derived CARs (34–36). CD22, an alternative candidate for anti-CD19 CAR T cells, which is also expressed on most B-ALL cells. Recently, CD22 CAR-T immunotherapy has achieved similar anti-leukemia effects in patients with r/r B-ALL, including those who previously received CD19 CAR-T cells and had dim/negative expression of CD19, resulting in a CR rate of 73 - 80% (26, 27). Evidence from preclinical models of solid tumors has shown that dual CAR-T cells may exhibit synergistic effects, permitting the optimization of response rates compared with those achieved by targeting a single antigen (37, 38). CAR T cells with dual targeting of CD19 and CD22 have demonstrated promising clinical outcomes in the treatment of hematological cancers with low CD19 relapse rate (39–41). We performed a meta-analysis by synthesizing the current evidence to assess the efficacy and safety of anti-CD22 and anti-CD19/CD22 CAR-T cell therapies for hematological tumors.

A total of 10 clinical trials with 194 r/r B-ALL patients met the inclusion criteria of this meta-analysis. The overall complete response rates of CD22 and CD19/CD22 CAR-T cell therapies for relapsed or refractory B-ALL were 0.75 and 0.87, the pooled estimate of CR rate of CD19/CD22 bispecific CAR-T cell therapy was higher than that of CD19 targeted CAR-T cell therapy as reported in Meng et al.’s meta-analysis. It is inferred that dual antigen targeting may prevent recurrence, given that a single leukemia stem cell is unlikely to downregulate both CD19 and CD22 at the same time (28). Notably, MRD negative rates of CD19/CD22 dual targeted CAR-T cell therapy was significantly greater than that of anti-CD22 CAR-T cell therapy (p < 0.01). The assessment of MRD has been widely used for the definition of deepness of treatment response and the main prognostic factor for different hematological malignancies (42, 43). However, due to limited data of follow-up, relapse after CD19/CD22 CAR-T cell treatment remains to be investigated in the upcoming clinical trials. CRS and neurotoxicity, also known as immune effector cell associated neurotoxicity syndrome, are considered to be the main obstacles to the widespread of CAR-T cell therapy (44). In this meta-analysis, the pooled CRS rates of CD22 targeted and CD19/CD22 targeted immunotherapy reached 0.92 and 0.94, a high fraction of the which was Grade 1 and 2 CRS. Likewise, mild or moderate neurotoxicity were observed in the studies included. In the investigation of Pan et al. Grade 2 neurotoxicity as manifested by brief generalized seizure occurred on day 7 after CAR T-cell treatment and was immediately managed with mannitol, furosemide, and dexamethasone, and was completely resolved within 3 days. Furthermore, low percentage of severe CRS (≥ Grade 3) was observed in several included studies. Prolonged cytopenia, B cell aplasia, and heightened infection risks were problems with dual CAR platforms that should be investigated and addressed in the future (45). In addition, the financial issue of CAR-T cell therapy could not be neglected in the clinical setting, in the United States, for example, with a price of $475000 for the pediatric CAR-T product, major payers have struggled to set adequate hospital reimbursement, which may lead to delays in care that could affect child health outcomes (46). It highlights the need to explicitly consider value when setting prices for treatments with CAR-T.

This is the most recent meta-analysis by far which investigated the efficacy and safety of CD22-specific and CD19/CD22-bispecific CAR-T cell therapy in patients with hematologic malignancies. This study was carried out under the guidance of PRISMA. Database search, study selection, data extraction, and quality assessment were performed by two independent reviewers to minimize potential bias. Heterogeneity and publication bias were appraised using statistical and graphical approaches. Mild to moderate heterogeneities and insignificant publication bias were detected in the current study.

There are limitations in this study. Although 10 studies were included in this study, the overall sample size remained small, the interpretation of the results in the meta-analysis should be with caution. Neither meta regression nor subgroup analysis was performed because of insufficient number of studies in each subgroup. Furthermore, due to limited number of studies, data on overall survival and progression-free survival in relapsed/refractory B-ALL patients treated with CD22 targeted or CD19/CD22 targeted CAR-T immunotherapies could not be synthesized at present. Thus, narrative depiction was proposed based on the available information retrieved from enrolled studies. Furthermore, in this study, adverse events regarding CRS and neurotoxicity were evaluated, other related adverse events including abnormal hemogram indices and infection were not pooled up which are actually of great clinical significance in the real-world practice. There is considerable heterogeneity as CRS may not be graded per a single criterion in all included studies, more well-designed clinical trials with larger sample sizes investigating the adverse events, especially on prolonged cytopenia, B cell aplasia, and heightened infection risks, are warranted. It’s reported that delayed immune reconstitution is the major issue behind diminished SARS-CoV-2 vaccine responses and increased infections, and this risk is potentially compounded by dual antigen targeting (47, 48).

## Conclusion

Anti-CD22 alone and CD19/CD22 bispecific targeted CAR-T cell immunotherapies displayed deep and durable responses and manifested manageable safety profiles in patients with relapsed/refractory B-ALL. Studies with larger sample sizes, and prolonged follow-up are warranted for further evaluation of the efficacy and safety of CAR-T cell immunotherapy.

## Data availability statement

The original contributions presented in the study are included in the article/**Supplementary Material**. Further inquiries can be directed to the corresponding authors.

## Author contributions

LL, LW and RX contributed to the conception and design of the study. LL and LW searched the database. LL and ZW extracted data. LW conducted data analysis. LL and LW wrote the manuscript. QL, YZ, and RX revised the manuscript. All authors contributed to the article and approved the submitted version.
