# Supplementary material for: Efficacy and safety of CD22-specific and CD19/CD22-bispecific CAR-T cell therapy in patients with hematologic malignancies: A systematic review and meta-analysis
Source: Front Oncol. 2022 Dec 29;12:954345. doi: 10.3389/fonc.2022.954345 (PMC9837739; doi:10.3389/fonc.2022.954345)
Supplement: Supplementary file 1 [file Table_1.docx]

**Efficacy and Safety of CD22-specific and CD19/CD22-bispecific CAR-T Cell Therapy in Patients with Hematologic Malignancies: A Meta-analysis**

Lili Li, Luqin Wang, Qinhua Liu, Zhonghui Wu, Yulong Zhang, Ruixiang Xia

**Supplementary Table 1**. Results of quality assessment of included studies.

| **Study** | **Selection** | | | |  | **Comparability** |  | **Outcome** | | |  | **Quality score** |
| --- | --- | --- | --- | --- | --- | --- | --- | --- | --- | --- | --- | --- |
|  | **Representativeness of the exposed cohort** | **Selection of the nonexposed cohort** | **Ascertainment of exposure** | **Demonstration that outcome of interest was not present at start of study** |  | **Comparability of cohorts on the basis of the design or analysis** |  | **Assessment of outcome** | **Was follow-up long enough for outcome to occur** | **Adequacy of follow up of cohorts** |  |  |
| Pan 2019 | ★ | NA | ★ | ★ |  | ★★ |  | ★ | ★ | ★ |  | 8 |
| Dai 2020 | ★ | NA | ★ | ★ |  | ★ |  | ★ | ★ | ★ |  | 7 |
| Shah 2020 | ★ | NA | ★ | ★ |  | ★★ |  | ★ | ★ | ★ |  | 8 |
| Wang 2020 | ★ | NA | ★ | ★ |  | ★★ |  | ★ | ★ | ★ |  | 8 |
| Wang 2020 | ★ | NA | ★ | ★ |  | ★★ |  | ★ | ★ | ★ |  | 8 |
| Cordoba 2021 | ★ | NA | ★ | ★ |  | ★★ |  | ★ | ★ | ★ |  | 8 |
| Hu 2021 | ★ | NA | ★ | ★ |  | ★ |  | ★ | ★ | ★ |  | 7 |
| Liu 2021 | ★ | NA | ★ | ★ |  | ★ |  | ★ | ★ | ★ |  | 7 |
| Singh 2021 | ★ | NA | ★ | ★ |  | ★ |  | ★ | ★ | ★ |  | 7 |
| Spiegel 2021 | ★ | NA | ★ | ★ |  | ★★ |  | ★ | ★ | ★ |  | 8 |
| Tan 2021 | ★ | NA | ★ | ★ |  | ★★ |  | ★ | ★ | ★ |  | 8 |

NA, not applicable.
